# Quantitative Analysis of Heavy Metals and Organic Compounds in Soil from Deir Kanoun Ras El Ain Dump, Lebanon

**DOI:** 10.1155/2020/8151676

**Published:** 2020-05-26

**Authors:** Jamilah Borjac, Manal El Joumaa, Lobna Youssef, Rawan Kawach, Diane A. Blake

**Affiliations:** ^1^Department of Biological Sciences, Beirut Arab University, Debbieh, Lebanon; ^2^Department of Chemistry, Beirut Arab University, Debbieh, Lebanon; ^3^Department of Biochemistry and Molecular Biology, Tulane University School of Medicine, New Orleans, LA, USA

## Abstract

Recently, there has been a worldwide concern regarding soil contamination by heavy metals and organic compounds, especially in the developing countries including Lebanon that has suffered from solid waste mismanagement for decades. Deir Kanoun Ras El Ain is a village in southern Lebanon that possesses one of the country's worst dumps, and its leachates influx into a running canal that irrigates surrounding agricultural lands. The aim of this study was to determine the levels of some toxic heavy metals and organic compounds in different soil samples collected from the dump and along the canal during winter and summer seasons. Six research sites (four from the dump and two along the canal) were selected, and the soil samples for analysis were collected from a depth of around 10 cm. Heavy metals (lead, cadmium, arsenic, and mercury) and organic compounds (phthalates, bisphenol A, and polyaromatic hydrocarbons) content were determined using atomic absorption and high pressure liquid chromatography, respectively. The conducted research confirmed high levels of contamination in the collected soil samples by both heavy metals and organic compounds. The present study provided evidence that different sampling sites accumulated heavy metals at concentrations that exceeded the average maximum permissible levels for sewage sludge and agricultural land. These findings suggest the need for mitigation measures by the Lebanese authorities and new waste management programs to resolve the problems associated with uncontrolled dumping of solid wastes in Lebanon.

## 1. Introduction

Recently, environmental contamination by high levels of heavy metals and organic compounds has raised the global public health concerns especially in the developing countries [1]. The major toxic heavy metals that lead to soil pollution are copper (Cu), zinc (Zn), cadmium (Cd), lead (Pb), arsenic (As), and mercury (Hg) [2, 3]. Unlike organic pollutants, which are ultimately degraded to carbon dioxide and water, heavy metals, once discharged into the environment, are difficult to remove. These metals bind to soils and sediments and persist in the environment until mobilized by changes in weather patterns, hydrogeology, and/or the overlying vegetation [4]. Besides, contaminated soils appear to act as a sink for organic compounds such as phthalates, bisphenols, and polycyclic aromatic hydrocarbons (PAHs) which originate from the dumping/burning of industrial, household, electronic, plastic, and medical wastes [5]. In the soil, these organic compounds have low mobility, high durability, and high affinity to soil organic matter [6]. It has been reported as well that, at dumpsites where solid wastes are burned, contamination by heavy metals is often accompanied by contamination by organic compounds [7, 8].

In addition, soil contamination by heavy metals and organic compounds may affect water resources and agricultural areas where the soil-crop system plays a chief role in the exposure of humans to contaminants [9, 10]. Humans may be exposed to soil contaminants through the food chain where consumption of crops grown in contaminated soils results in tissue accumulation and significant toxicity and illness when reaching critical values [11, 12]. For example, chronic exposure to high levels of heavy metals has been associated with higher risks of bladder, kidney, liver, lung, and skin cancer [13]. More recently, toxic metal exposure has also been associated with an increased risk of cardiovascular disease [14]. Moreover, organic compounds, especially PAHs, have been shown to exert carcinogenic, mutagenic, immunotoxic, and endocrine disrupting effects [15].

Lebanon has suffered from solid waste mismanagement for decades [16]. It lacks solid waste management plans, which resulted in random disposal, dumping, and open burning of wastes across the country [17]. Hundreds of landfills exist across the entire country and the leachates from these landfills influx into the soil and water resources [18]. The lax environmental policies in Lebanon resulted in exacerbated waste problems and detrimental effects on the health of residents [19]. Deir Kanoun Ras El Ain is one of a number of Lebanese villages where solid waste had been mismanaged for many years. It harbors a dump that contains medical, industrial, and household wastes, and its leachates influx into a running canal that irrigates surrounding agricultural lands [20]. The present study was conducted to assess the levels of different heavy metals and organic compounds in soil samples collected from the Deir Kanoun dump and canal at different sites, and during winter and summer seasons of 2017.

## 2. Materials and Methods

### 2.1. Site and Sample Collection

Samples were collected in triplicate during winter and summer seasons of 2017. Sampling sites included two along the canal and four surrounding the dump as presented in Table 1. The soil samples were collected from topsoil at a depth of 0–10 cm and stored in sterile bags. The soil samples were air-dried at room temperature prior to the analysis of heavy metals, phthalates, bisphenol A, and PAHs.

### 2.2. Heavy Metals Analysis

The concentrations of heavy metals such as lead, cadmium, arsenic, and mercury (Pb, Cd, As, and Hg) were assessed in soil samples using atomic absorption spectrophotometer (Bioteckno, Model: GF95Z, UK). Analysis was performed at the Lebanese Agricultural Research Institute (LARI) in Lebanon. Details of the analysis procedure are available in Supplemental Materials.

### 2.3. Organic Compounds Extraction and Analysis

The methods for phthalates and bisphenol A extraction and fractionation were carried out according to those described by Fromme et al. [21]. Methods for polycyclic aromatic hydrocarbon extraction and fractionation were as described by Pule et al. [22]. Details are available in Supplemental Materials.

## 3. Results

### 3.1. Heavy Metals

**Figure 1:**
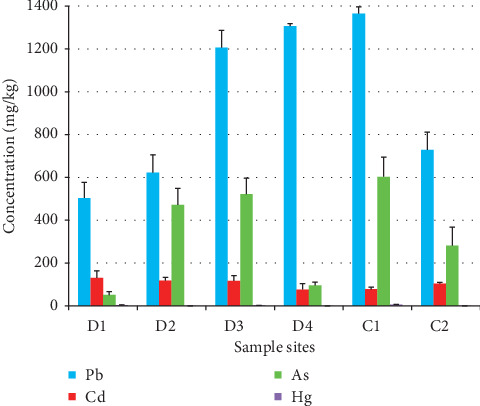
Distribution of heavy metals among different sampling sites.

The concentrations of heavy metals assessed in soil samples collected during summer season are presented in Table 2, and the soil quality standards used to evaluate the levels of contamination are presented in Tables 3 and 4. The variation in heavy metal concentrations in the soils at the studied sites is shown graphically in Figure 1.

Pb, Cd, As, and Hg were abundant in all of the collected samples from dump and canal, where their concentrations ranged 504.3–1365 mg/kg, 77–131.1 mg/kg, 51–603.3 mg/kg, and 0.16–6.48 mg/kg, respectively. Pb, As, and Hg concentrations were the highest in soil samples collected from C1 site. Concerning samples collected around the dump, D4 site contained the highest concentrations of Pb (1306.07 mg/kg), while Cd and Hg were at the highest levels at D1 site (131.1 mg/kg and 3.58 mg/kg, resp.). As was highly abundant in soil samples collected from D2 (471.97 mg/kg) and D3 (521.7 mg/kg) sites.

**Figure 2:**
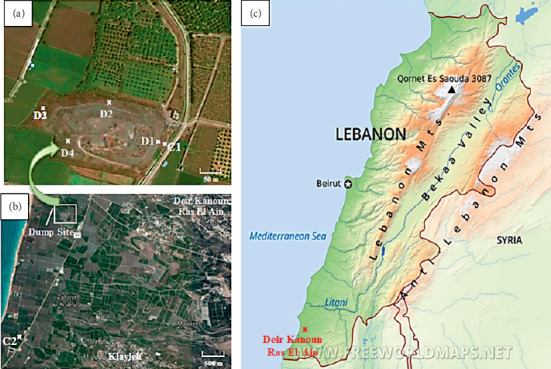
(a, b) The study area showing the dump and canal sites (D1, D2, D3, D4, C1, and C2). (c) Map of Lebanon showing Deir Kanoun Ras El Ain village.

While absolute values of heavy metal contamination are important, especially during remediation efforts, these values do not take into account the relative toxicity of the individual metals present at each site. These relative toxicities are considered in the determination of the maximum permission limits (MPL) set by various governmental regulatory agencies. The maximal permissible levels for Pb, Cd, As, and Hg in sewage sludge are shown in Table 3. These MPLs are designed to restrict the use of heavily contaminated sewage sludge as agricultural fertilizer [32]. Since much of the land immediately adjoining this dump and canal is under cultivation (see Figure 2), we also report the MPL for heavy metals in agricultural soil in Table 4.

Comparison of the levels of heavy metals to the sewage sludge MPLs promulgated by several countries showed that Pb levels at D3, D4, and C1 sites surpassed the quality standards of EEC, France, US EPA, and CCME. In addition, the levels of Cd at all sites except D4 and C1 surpassed the sewage sludge MPLs of EEC, France, US EPA, and CCME. The D4 and C1 sites had Cd levels that fell below the MPLs promulgated by the US EPA; however, these Cd levels exceeded the Cd MPLS set by the European Union, Canada, and France. The levels of As at all sites except D1 highly exceeded the US EPA and CCME sewage sludge standards. Only three EU member states (Bulgaria, Czech Republic, and Denmark) currently regulate As in sewage sludge, with an MPL of 25–30 mg/kg [32]; the levels of As at all sites exceeded this value. Levels of Hg were below the MPL values of all sewage sludge guidelines.

**Figure 3:**
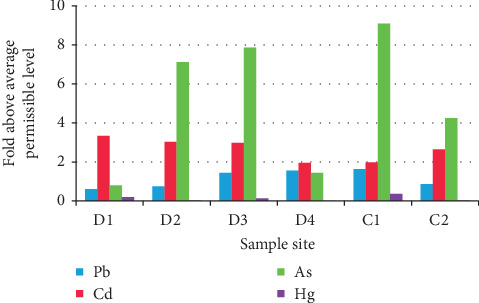
Relative toxic effects of heavy metals at different sampling sites, expressed as fold above average permissible level in sewage sludge.

An average MPL value for each metal was calculated (Table 1 supplementary material) and used to calculate the ‘fold above the average maximum permissible level' for each metal at each site, as shown in Figure 3. This analysis provides insight into the relative toxicity of the tested metals at each site. Results show that As levels had the highest fold above average MPL at most sites, followed by Cd and Pb. Samples from site C1 were the most concerning, with the following values: As, 9.5-fold; Cd, 2-fold; and Pb, 1.63-fold above the maximum average permissible level of heavy metals.

Soil samples collected were also from surrounding agricultural lands apart the sludge soil. Thus, we compared the levels of the heavy metals analyzed in these samples to the maximum permissible limits of agricultural soil, as set by the guidelines shown in Table 4. These comparisons showed that the concentrations of Pb, Cd, and As in all soil samples surpassed the quality standards of FAO/WHO, European council/union, United States, France, Germany, Austria, China, and Arab-German Cooperation project 1997–2003. The level of analyzed Hg was above the MPLs only at D1 and D3 site. Note that site C1, which has relatively high levels of heavy metals and is directly in contact with the dump leachate, is not used for planting purposes.

**Figure 4:**
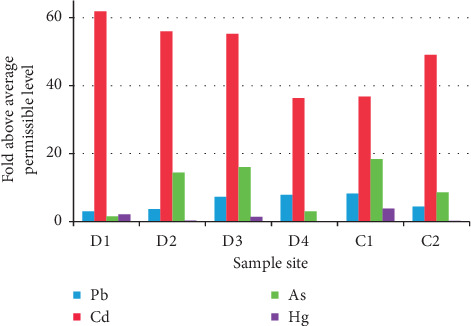
The distribution of fold above average permissible level of different heavy metals for agricultural soil among different sample sites.

To provide a clearer indication of which of the metals may pose the greatest risks to residents who may consume food grown in the agricultural land surrounding the studied dump area, the fold above the agricultural MPL for each metal at each site was also calculated and presented in Figure 4. Results showed that Cd levels highly exceeded the average MPL, followed by As and Pb. The highest fold above MPL for Cd was obtained at site D1 (62 -fold), whereas the highest fold above MPL for As, Pb, and Hg was obtained at site C1 (18.4-, 8.2-, and 3.8-fold, resp.).

### 3.2. Organic Compounds

The levels of organic compounds expected at dumpsites (plastic residues and products of incomplete combustion) were also assessed in soil samples collected from the dump and canal sites during two seasons. These data are presented in Table 5.

As seen in Table 5, winter collections (W) showed that samples collected from the D1 site contained the highest concentrations of di-n-butyl-phalate (DBH. 6.25 mg/kg) and bis(2-ethylhexyl) phthalate (DEHP, 12.59 mg/kg) and also of bisphenol A that leach from the food storage containers, bottle tops, and the coatings on medical devices (BPA, 1.454 mg/kg). The concentrations of DEHP and BPA showed a decreasing pattern from the D1 to the D4 sites. Benzo *α*-pyrene (BAP), a carcinogenic polycyclic hydrocarbon produced during incomplete combustion, was detected only in samples collected from the D3 (0.022 mg/kg) and D4 (0.012 mg/kg) sites. Samples collected from C2 site showed detectable concentrations of BAP (0.075 mg/kg). Summer collections (S) revealed a similar abundance of residues from plastic waste among different sites. DBH, DEHP, and BPA concentrations showed decreasing patterns where the highest concentrations were in samples collected from D1 site (8.24, 15.56, and 2.027 mg/kg, respectively) while the lowest concentrations were in samples collected from D4 site (DBH was undetectable; DEHP and BPA were 1.84 and 0.024 mg/kg, respectively). Regarding soil samples collected near the canal, DBH concentrations in samples collected from C2 site (1.79 mg/kg) were higher than the ones collected from C1 site (1.23 mg/kg). Likewise, DEHP and BPA concentrations in C2 site (5.04 and 0.354 mg/kg, respectively) were higher than those from C1 site (3.16 and 0.064 mg/kg). Benzo *α*-pyrene was undetectable in all samples collected during the summer season.

## 4. Discussion

The lack of waste management policies in Lebanon has led to uncontrolled dumping of both household and hazardous solid wastes [16–19]. Deir Kanoun Ras El Ain is one of the villages that suffered from the presence of a dump that includes medical, industrial, and household wastes. Leachates from this dump flow into a canal that circles the dumpsite. These wastes and their leachates are major sources of heavy metals and organic compounds that are known to cause adverse health and environmental hazards [20].

Regarding heavy metals, our findings indicate that Pb, Cd, and As were the most serious metal pollutants in soil samples collected from Deir Kanoun dump and canal. Upon comparing our data to previously published research studies, it was noted that our findings are similar to those obtained by Tang et al. [33] where there were high levels of Pb and Hg in soil samples collected from a dumpsite in China (81.3–2374.1 mg/kg and 0.2–3.2 mg/kg, respectively). Similarly, the levels of Pb and Hg in our study are similar to those obtained in soil samples collected from a dumpsite in Croatia (605–968 mg/kg and 0.73–1.51 mg/kg, resp.) [34]. However, our obtained levels of Cd are higher than those of China's dumps (0.6–12.5 mg/kg) [33], Nigeria (17.00–47.06 mg/kg) [35], and Croatia (70–72 mg/kg) [34]. Likewise, Pb levels in this study are higher than those collected from dumps in Nigeria (63.58–418.58 mg/kg) [35] and in Sweden (254–895 mg/kg) [36].

Additionally, the levels of Pb, Cd, and As exceeded the sewage sludge maximum permissible limits (MPL) of several quality standards. Fold above average MPL for each metal at each site showed that As levels had the highest fold above average MPL followed by Cd and Pb. Comparing our results to the MPL of heavy metals in agricultural soil set by different guidelines showed that the concentrations of Pb, Cd, and As in all soil samples greatly surpassed the quality standards of several agencies. It should be noted that the dump area in Deir Kanoun Ras El Ain is surrounded by agricultural lands, and the canal from which samples were collected irrigates these lands. The fact that Cd and As greatly exceed the MPLs for both sewage sludge and agricultural soil indicates that these two metals may pose a risk to residents who live near the dumpsite and consume crops grown in contaminated agricultural lands. In fact, high levels of Pb, Cd, and As have been shown to exert damaging effects and serious disorders in living organisms [37, 38]. For example, short term exposure to Pb at high concentrations often causes vomiting, fatigue, diarrhea, convulsions, coma, or death [39], and even low concentrations of this metal are dangerous to young children because it can affect their nervous systems [40]. Inhabitants exposed to Cd may develop damage in their lungs, bones, and kidneys [41]. In humans, even low levels of As are considered toxic as they may cause adverse damaging effects on the brain, liver, kidney, stomach, and intestines [42, 43].

Moreover, the findings reported herein are of serious concern since Cd has been widely associated with decreased bone mineral density which subsequently leads to increased risk of bone fracture in both experimental models and populations-based studies [44, 45]. This fact led us to investigate the prevalence of bone fracture among Lebanese and Syrian refuges living in three villages surrounding the dump region. Unpublished results of data collected from residents of the region under study revealed that 27.4% of participants suffered bone fractures. Such findings are consistent with previous studies that proved the associations between low-level Cd exposure and (1) decreased bone mineral density and (2) increased risk of bone fractures in elderly men [46–50].

In addition to heavy metals, we were also able to detect the presence of phthalates (DBP and DEHP), bisphenol A, and benzo(*α*)pyrene in most of the collected soil samples. Regarding phthalates, a study by Zorníková & Hřivna in Czech Republic showed that agricultural soil samples contained 0.28–1.59 mg/kg di-n-butyl phthalate (DEHP) and 0.03–0.73 mg/kg bis(2-ethylhexyl) phthalate (DBP) which are lower than the findings of the present study [51]. In addition, a study by Vikelsøe et al. in Denmark revealed that the concentrations of di-(n-butyl)-phthalate (DBP) and di-(2-ethylhexyl)-phthalate (DEHP) in sewage sludge soil samples were 439–453 *μ*g/kg and 1110–1900 *μ*g/kg, respectively [52]. On the other hand, the concentrations of DBP and DEHP in agricultural soil samples were 0.5–1.1 *μ*g/kg and 12–40 *μ*g/kg, respectively. According to the Danish guidelines, the levels of DBP and DEHP should be limited to 0.01–0.98 *μ*g/kg in fertilized soils and 1450–2430 *μ*g/kg in sewage sludge [53]. Most of our findings exceed the limits set by the Danish guidelines suggesting risks for organisms and humans especially that different studies documented the ability of DBP and DEHP to act as endocrine disruptors [54–56].

Besides phthalates, bishenol A (BPA) is an important organic compound that acts as an endocrine disruptor, mainly as an estrogen, and it is widely used in the industrial manufacture of plastics [57]. Due to the substantial widespread of the use and involvement of BPA in soil amendments, there is a growing concern about its presence and possible toxicity in soils especially those cultivated with crops [58]. Our findings showed that the collected soil samples contained a range of 0.024–2 mg/kg of BPA which is greater than that found in sludge from the Midwest USA (4.6 mg/kg; [59]) and Canada (36.7 mg/kg; [60]). However, our findings are similar to those reported in several countries, such as Greece (0.62 mg/kg; [61]), Spain (0.005–0.68 mg/kg; [62]), South California (0.066–0.217 ng g−1; [63]); and Germany (0.004–1.363 mg/kg; [21]). On the other hand, they are higher than those reported in Gran Canaria, Spain (0.0014–0.055 mg/kg; [64]), and Ontario, Canada (∼0.004 mg/kg; [65]). Thus, our data imply that the levels of BPA in the samples collected in the current study are of a moderate level and within previously reported ranges.

Regarding PAHs, our study focused on benzo(*α*)pyrene which is one of the highly toxic PAHs in the soil-crop system [66]. It is released to the environment as a result of combustion and can easily accumulate in organisms exposed to it causing potential carcinogenic and mutagenic effects [67, 68]. Our results revealed the presence of benzo(*α*)pyrene in three sites only at concentrations of 0.012–0.075 mg/kg, which are similar to those reported in soil samples from regions in Poland (0.05–0.19 mg/kg; [69]), Spain (0.018–0.1 mg/kg; [70]), South Korea (0.0124–0.0211 mg/kg; [71]), and Canada (∼0.031 mg/kg; [72]). Also, our findings are similar to those obtained in soil samples collected from an E-waste dumpsite in China (0.0011–0.0256 mg/kg; [33]).

Finally, it is crucial to provide a brief comparison of pollution between the different sites of soil collection. Among all the studied sites, sites C1, D4, and D3 were the most heavily contaminated with heavy metals, while site D1 had the highest levels of organic compounds. There are several factors that lead to the different levels and distribution of heavy metals and organic compounds, such as the type of dumped waste at each site, soil properties, soil organic matter (SOM), distance to the sources of contamination, use of lands, type of grown crops, and dry or wet depositions which may explain why our summer collections had higher levels of organic compounds than winter collections [2, 3]. Moreover, site C2, which is farther from the dump, had higher levels of organic compounds than site C1. This observation could be attributed to the litter of consumer waste products and plastics at site C2 where refugee families live in camps with poor hygiene conditions and with little or no consideration to health protection. The high levels of heavy metals and organic compounds at this particular site imply that the individuals living in that region are at high health risks, knowing that the dump leachates influx to the canal used for irrigating the surrounding agricultural lands and drinking. However, data regarding human exposure are still limited and further studies are required to assess the impacts on human health.

It should be taken into consideration that the toxicological effects of such compounds depend on their bioavailability, metabolism, and excretion into organisms and humans. Thus, the current results suggest further investigation of the levels of heavy metals and organic compounds in crops grown in agricultural lands near the dump and irrigated from the canal. Furthermore, serious actions and mitigation measures must be taken by the Lebanese authorities to solve the current environmental disaster as well as other similar dumps. Additionally, it is hoped that these findings lead to a greater awareness of the hazardous consequences of unregulated waste dumping.

## 5. Conclusion

The results of the current study show that soil samples collected from Deir Kanoun Ras El Ein dump and canal were heavily contaminated with heavy metals. The presence of organic compounds including phthalates, bisphenol, and PAHs was also noted. The concentrations of heavy metals highly exceeded the permissible limits recommended by different agencies. The presence of heavy metals and organic compounds in the irrigation canal may also affect agricultural lands that are irrigated by canal's water and, ultimately, the health of inhabitants who consume the crops grown in these contaminated soils. More studies are clearly needed to determine the extent of heavy metal contamination at various distances from the dump's site and in the crops grown in fields both adjacent to and more distant from this dump. Since both the heavy metals and organic pollutants are a result of the uncontrolled dumping of solid waste at this site, stricter environmental policies regarding waste management should be implemented by the Lebanese authorities and guidance should be given to local inhabitants about healthy management practices regarding information pertinent to their health status.

## Figures and Tables

**Table 1:** Sampling sites from Deir Kanoun dump and canal.

Site	Location details
C1	(i) Deir Kanoun canal
	(ii) **Latitude (N):** 33^o^ 13′ 43.77″; **longitude (E):** 35^o^ 15′ 48.061″
C2	(i) Klayleh
	(ii) **Latitude (N):** 33^o^ 28′ 48.989″; **longitude (E):** 35^o^ 20′ 20.399″
D1	(i) Dump contact with C1
	(ii) **Latitude (N):** 33^o^ 13′ 43.77″; **longitude (E):** 35^o^ 15′ 48.061″
D2	(i) Dump ∼ 90^o^ to C1
	(ii) **Latitude (N):** 33^o^ 11′ 36.596″; **longitude (E):** 35^o^ 13′ 27.829″
D3	(i) Dump ∼ 180^o^ to C1
	(ii) **Latitude (N):** 33^o^ 13′ 122.609″; **longitude (E):** 35^o^ 12′ 57.16″
D4	(i) Dump ∼ 220^o^ to C1
	(ii) **Latitude (N):** 33^o^ 13′ 43.453″; **longitude (E):** 35^o^ 13′ 33.628″

**Table 2:** Average concentrations (*n* = 3) of heavy metals in soil collected from Deir Kanoun dump and canal. Contaminant concentrations of all metals are expressed in mg/kg.

Heavy metal	Sample site					
	D1	D2	D3	D4	C1	C2
Pb	504.3 ± 73.02	622.1 ± 82.98	1206.1 ± 80.35	1306.1 ± 12.62	1365 ± 31.5	728.4 ± 83.68
Cd	131.1 ± 32.21	118.7 ± 15.03	117 ± 24.44	77 ± 27.22	78 ± 9.46	104.4 ± 5.5
As	51 ± 15.54	471.97 ± 77.42	521.7 ± 74.68	95.87 ± 15.75	603.3 ± 91.08	281.6 ± 86.81
Hg	3.587 ± 1.27	0.459 ± 0.156	2.39 ± 0.416	0.16 ± 0.044	6.48 ± 0.761	0.38 ± 0.073

**Table 3:** Guidelines for the maximum permissible limit (MPL) values of selected heavy metals in sewage sludge used as agricultural fertilizer.

Heavy metal	US EPA^a^ mg/kg	CCME^b^ mg/kg	EEC^c^ mg/kg	France^d^ mg/kg	Average^*∗*^ value mg/kg
Pb	840	600	750–1200	800	804
Cd	85	22	20–40	20	39.25
As	75	40–75	-	-	66.25
Hg	17	24	16–25	10	17.88

^a^United States Environmental Protection Agency [23]. ^b^Canadian Council of Ministers of the Environment [24]. ^c^European Economic Community/European Union [25]. ^d^Data for France [25]. ^*∗*^Where a range is reported, the average value was calculated from the midpoint.

**Table 4:** Guidelines for the maximum permissible limit (MPL) values of selected heavy metals in agricultural soil.

Metal	FAO/WHO^a^ mg/kg	EC^b^mg/kg	US^c^mg/kg	France^d^ mg/kg	Germany^d^ mg/kg	Austria^d^ mg/kg	SEPA China (Grade III) mg/kg	Arab-German Coop Project^f^mg/kg	Average^*∗*^ mg/kg
Pb	90–400	50–300	50–300	70–150	100	100	500	100	166.25
Cd		1–3	1.6	1–3	1.5–3	5	1	1	2.12
As	-	20	14	30	50	50	-	-	32.8
Hg	1	1	0.5	1	2	5	1.5	-	1.71

^a^Food and Agriculture Organization of the United Nations and the World Health Organization [26]. ^b^European Council/Union [27]. ^c^United States [28]. ^d^Data for France, Germany and Austria [29]. ^e^State Environmental Protection Administration of China [30]. ^f^Arab-German Cooperation project 1997–2003 [31]. ^*∗*^Where a range is reported, the average value was calculated from the midpoint.

**Table 5:** Average concentrations (*n* = 3) of organic compounds in soil samples collected from Deir Kanoun dump and canal during winter and summer seasons. Contaminant concentrations are expressed in mg/kg.

Organic compound	Sample site										
	D1		D2		D3		D4		C1	C2	
	W	S	W	S	W	S	W	S	S	W	S
Di-n-butyl phthalate	6.25 ± 0.002	8.24 ± 0.003	—	1.27 ± 0.0003	2.13 ± 0.001	2.42 ± 0.001	—	—	1.23 ± 0.002	—	1.79 ± 0.003
Bis(2-ethylhexyl) phthalate	12.59 ± 0.003	15.56 ± 0.004	8.96 ± 0.004	11.13 ± 0.002	3.3 ± 0.002	4.055 ± 0.003	—	1.84 ± 0.001	3.16 ± 0.002	—	5.04 ± 0.004
Bisphenol A	1.454 ± 0.0002	2.027 ± 0.001	0.888 ± 0.0002	1.23 ± 0.002	0.032 ± 0.0003	0.049 ± 0.0001	—	0.024 ± 0.0002	0.064 ± 0.0001	—	0.354 ± 0.0001
Benzo *α*-pyrene	—	—	—	—	0.022 ± 0.0002	—	0.012 ± 0.0005	—	—	0.075 ± 0.0003	—

(—) means not detectable.

## Data Availability

All data in this study are included in the figures, tables, and supplementary data.

## Supplementary Materials

Supplementary Materials It includes detailed methods to determine the heavy metals and organic compounds and our calculation of average minimum permissible levels of heavy metals and their relationships to levels in the soils analyzed in this study, as well as graphic of organic contaminant levels at test sites during winter and summer.
