# Supplementary material for: Quantitative Analysis of Heavy Metals and Organic Compounds in Soil from Deir Kanoun Ras El Ain Dump, Lebanon
Source: ScientificWorldJournal. 2020 May 26;2020:8151676. doi: 10.1155/2020/8151676 (PMC7271233; doi:10.1155/2020/8151676)
Supplement: Supplementary Materials — It includes detailed methods to determine the heavy metals and organic compounds and our calculation of average minimum permissible levels of heavy metals and their relationships to levels in the soils analyzed in this study, as well as graphic of organic contaminant levels at test sites during winter and summer. [file 8151676.f1.docx]

**Supplemental Material for**

**Analysis of Metals in Soil around an Unregulated Dump in South Lebanon Reveals Cadmium and Arsenic Levels that May Pose Risks to Nearby Residents**

J. Borjac, M. El Joumaa, L. Youssef, R. Kawach, D. A. Blake

**Table of Contents Page Number**

**Material and Methods . . . . . . . .** 2-3

***Heavy Metals Analysis . . . . . . .*** 2

***Analysis of Organic Contaminants . . . . . .*** 2-3

**Results**

**Calculation of average Minimum Permissible Levels of heavy metals**

**and relationships to levels in the soils analyzed in this study . . .** 4

**Graphic of organic contaminant levels at test sites, Winter and Summer. . 5**

**Materials and Methods**

**Heavy Metals analysis**. Soil (0.5 g) was digested in concentrated HNO_3_ (5 ml) in a basic reflux setup consisting of a heating mantle, a round bottom flask, a condenser. Samples were refluxed for 10 min at 95^o^C. After reflux, samples were cooled and additional 2.5 ml of conc. HNO_3_ were added. Reflux was continued until brown fumes were no longer visible. The solutions were then left to evaporate to approximately 5 ml at 95˚C. After cooling, 1 ml of water and 3 ml of 30% H_2_O_2_ were added to the solution with slow heating. The vessel was then cooled and 10 ml of 30% H_2_O_2_ were added in 1ml aliquots. This step was repeated until no changes in the appearance of the digested sample were observed. Samples were then heated at 95˚C for one hour followed by the addition of 5ml of conc. HCl. Samples were then covered with a watch glass and placed on a hotplate at 95˚C for 15 min. After cooling, samples were diluted to 50 ml with ddH_2_O and stored refrigerated. Solutions were analyzed for trace metals by atomic absorption spectroscopy. Final concentrations of the metals were calculated according to the following formula:

$$Concentration \left( \frac{mg}{Kg} \right)=\frac{Concentration \left( \frac{mg}{L} \right)\times V}{W}$$

where V is the final volume of solution, and W is the initial weight of sample measured.

**Analysis of organic compounds.** Freeze-dried soil samples (10 grams) were placed in Soxhlet extraction apparatus with either 200 mL cyclohexane/ethyl acetate (50:50) or methanol (for bisphenol analysis only) as extracting solvents, and they were left for 10 hours. Volume was reduced to 10 mL by rotary evaporation. Samples were reconstituted with n-hexane for GC analysis and with HPLC grade mobile phase for LC analysis^21^. The general methods for phthalates and bisphenol A extraction were according the method described by Fromme et al^21^. ***Phthalate and Bisphenol A fractionation.*** After extraction, phthalates were fractionated by HPLC using an ODS-C18 (25 cm x 4.6 mm, 5 μm) column. The used mobile phase consisted of acetonitrile:water at a ratio of 75:25 (v/v) at a flow rate 1 mL/min. Absorbance of the eluted compounds was measured at 226 nm^22^. Bisphenol A fractionation was performed by HPLC-UV analysis using Nucleosil-C18 (25 cm x 4.6 mm, 5 μm) column and acetonitrile:water (50:50, v/v) as a mobile phase. Flow rate was at 1 mL/min and the absorbance of the eluted products was measured at 275 nm^23^.

***Polycyclic aromatic hydrocarbon (PAH) extraction and fractionation.*** The extraction of PAHs was performed according to the method of Pule *et al.*^24^ Compounds were extracted with hexane/acetone (4:1) followed by sonication for 3 minutes (this procedure was repeated three times). The extracts were combined, centrifuged at 4,000 rpm for 5 minutes, and samples were concentrated with acetonitrile on a rotary evaporator prior to HPLC/UHPLC analysis^24^. HPLC-UV analysis of PAHs was performed using Agilent Supelco Machine coupled to a diode array detector and C18 (25 cm x 4.6 mm, 5 μm) column. Acetonitrile: water (70:30, v/v) was used as a mobile phase at a flow rate of 0.8 mL/min. Absorbance of eluted products was monitored at 220 and 254 nm^25^.

**
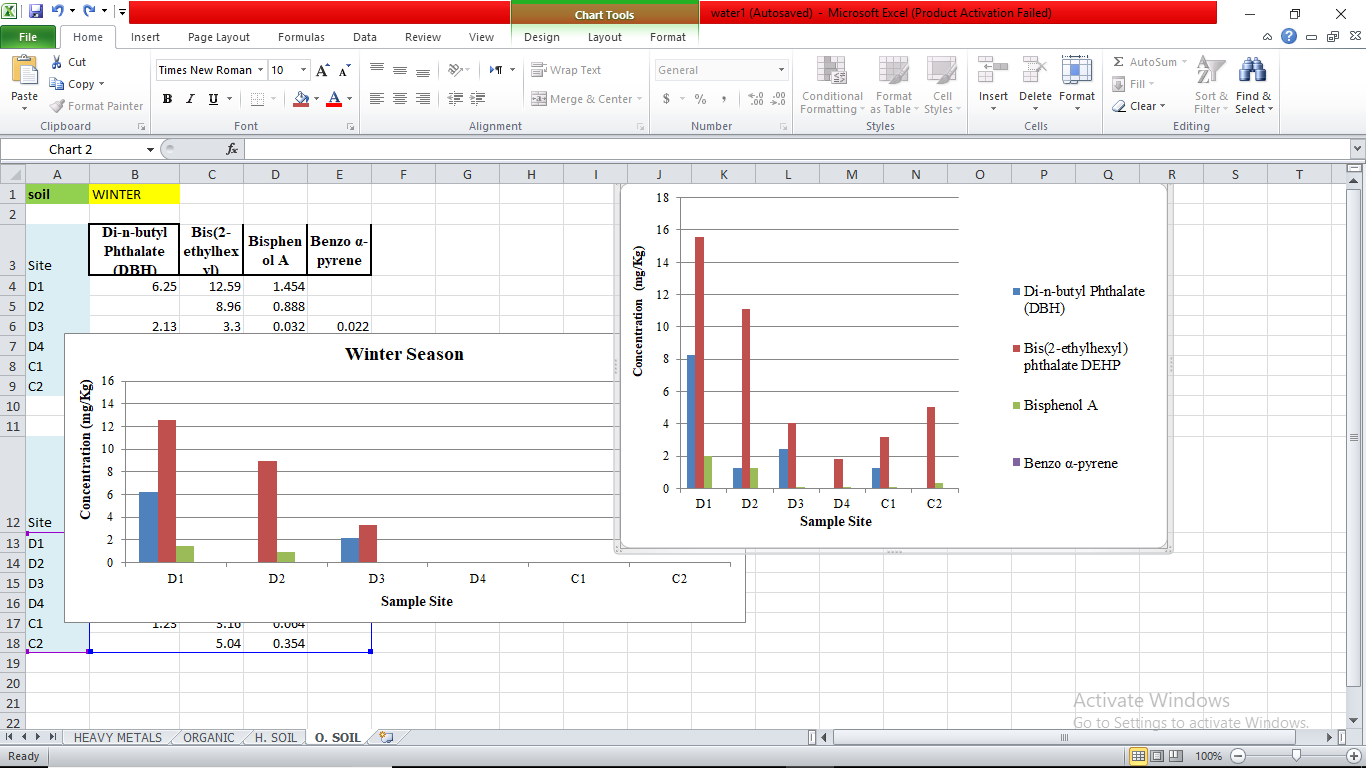
**

**
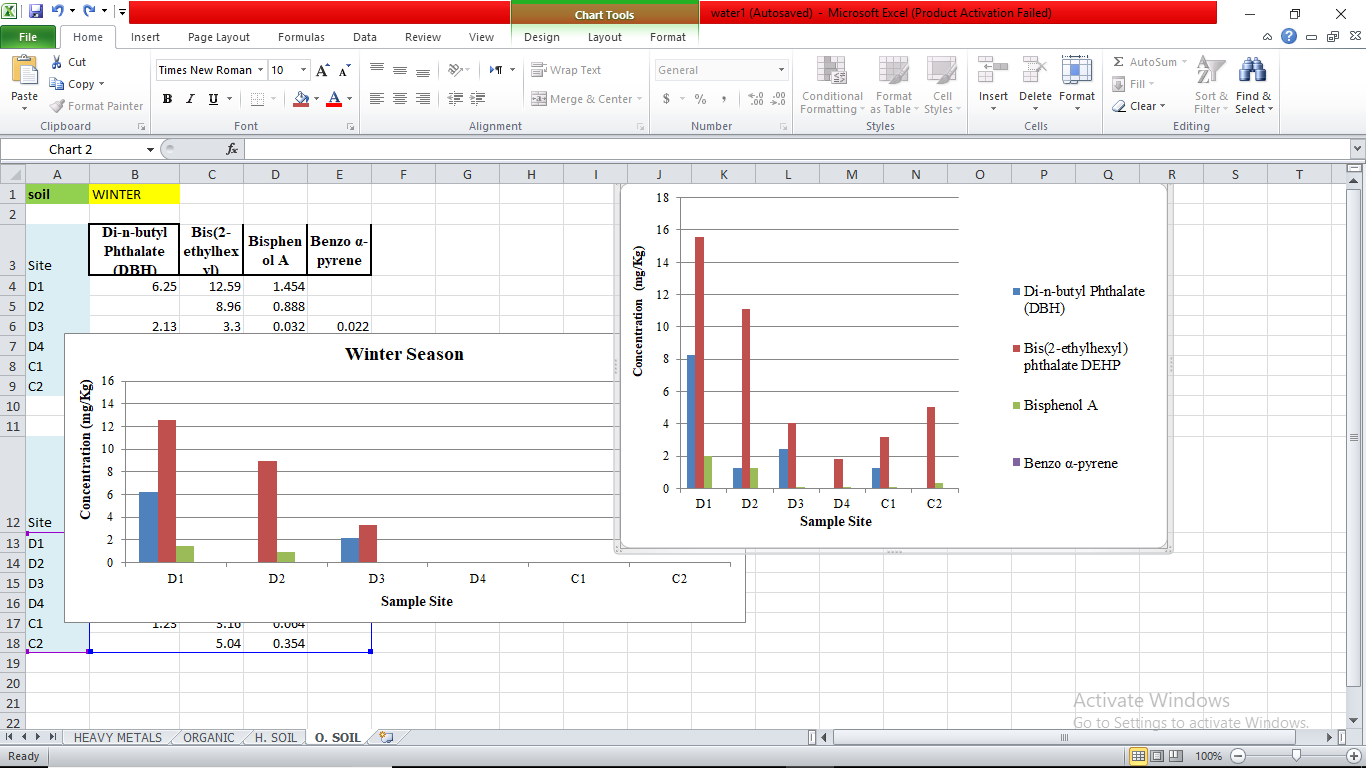
**

**Figure S1.** Distribution of different organic compounds among different sites.
